# Use of BioFire FilmArray gastrointestinal PCR panel associated with reductions in antibiotic use, time to optimal antibiotics, and length of stay

**DOI:** 10.1186/s12876-020-01394-w

**Published:** 2020-07-29

**Authors:** Daisy Torres-Miranda, Hana Akselrod, Ryan Karsner, Alessandra Secco, Diana Silva-Cantillo, Marc O. Siegel, Afsoon D. Roberts, Gary L. Simon

**Affiliations:** Division of Infectious Diseases, George Washington University School of Medicine and Health Sciences, 2150 Pennsylvania Avenue NW, Washington, DC, 20037 USA

**Keywords:** Gastrointestinal, FilmArray, Multiplex PCR, Diarrhea, Antibiotics, Stewardship

## Abstract

**Background:**

Rapid and accurate diagnostic tools are needed for appropriate management of infectious diarrhea.

**Methods:**

We evaluated the impact of the introduction of rapid multiplex PCR testing using the FilmArray gastrointestinal (GI) panel (BioFire Diagnostics, LLC, Salt Lake City, UT) at our institution, and compared the results to those of standard stool cultures.

**Results:**

The most common pathogens detected by the FilmArray GI panel were *Clostridium difficile* (55.0%), *Campylobacter* species (20.9%), *Salmonella* species (12.4%), and *Shigella*/EIEC species (12.4%). Rates of reproducibility in stool culture for these pathogens ranged from 56.3 to 77.8%. Co-detection of two or more organisms was common (24.2%), most commonly involving EPEC, EAEC, ETEC, and STEC. The time from arrival in the Emergency Department to discharge or admission to the hospital was unchanged after the introduction of FilmArray GI panel, but length of hospital stay was shorter (3 vs. 7.5 days, *p* = 0.0002) for the FilmArray group. The time to empiric antibiotics did not differ significantly, but optimal antibiotics were started earlier after introduction of the FilmArray GI panel (hospital day 1 vs. 2, *p* < 0.0001). More patients were discharged without antibiotics after introduction of the FilmArray GI panel (14.0% vs. 4.5%; *p* < 0.001).

**Conclusion:**

Our results demonstrate that the FilmArray GI panel is an important tool for improving both patient care and antibiotic stewardship, despite the tendency for positive results with multiple pathogens.

## Background

Infectious gastroenteritis remains a common cause of morbidity and mortality worldwide [1–3]. In the United States, an estimated 179 million episodes of diarrheal illness are estimated to occur each year, resulting in nearly 500,000 hospitalizations and 5000 deaths. The economic burden of Emergency Department (ED) visits for diarrheal illnesses alone in the USA has been estimated at $580 million per year [4–7]. Stool cultures have been the standard diagnostic tool for determining the microbial etiology of suspected bacterial infectious diarrhea. However stool cultures are time consuming, costly, have low positive yield, and their use by physicians can be inconsistent [8–11]. Rapid and accurate diagnostic tools are needed for appropriate management of infectious gastroenteritis. Commercially available multiplex polymerase chain reaction (PCR) testing panels may improve patient care and hospital workflow by allowing clinicians to choose the appropriate antimicrobials, or to avoid them if they are not indicated, aiding antibiotic stewardship and reduction of harm such as infection with *C.lostridium difficile* [12–15].

The FilmArray Gastrointestinal (GI) PCR Panel (BioFire Diagnostics, Salt Lake City, UT, USA) received FDA approval in 2014 and can identify 22 bacterial, viral, and protozoan pathogens associated with gastroenteritis directly from stool samples within approximately 1 h [16]. The proposed advantage of this test is the rapid pathogen identification thereby expediting appropriate treatment, including initiation or discontinuation of therapeutic agents and isolation precautions [17]. Currently, the question of whether the use of this test leads to quantifiable improvements in patient care over that of standard stool cultures is unresolved [18, 19]. We evaluated the impact of adding FilmArray GI panel testing to the standard management of patients presenting to the Emergency Department (ED) with suspected infectious diarrhea over an 18-month observation period (12 months prior to the introduction of GI BioFire, and 6 months after). This was a retrospective chart review performed with the specific aims of defining the reproducibility of FilmArray GI panel results in comparison to standard stool cultures, as well as time to disposition from the emergency department, duration of hospitalization, duration of antibiotic use, and time to optimal therapy, in a real-world setting. This study helps establish the role of multiplex PCR in the management of patients in the ED and hospital settings, and should be of interest to health care practitioners and leaders seeking to make decisions regarding the use of these testing modalities.

## Methods

### Patient population and data collection

We reviewed the electronic medical records for 300 adults seen at the George Washington University Hospital (GWUH) ED for a diagnosis of infectious diarrhea (identified by ICD-9/ICD-10 codes) from July 2015 through December 2016. FilmArray GI panel testing became available in May 2016. Variables abstracted into the study database included the FilmArray GI panel and stool culture results, other microbiology results (e.g. blood cultures), ED arrival and disposition times, duration of hospitalization, initiation of empiric and of optimal therapy, duration of final prescribed therapy, and outcome of the hospitalization. “Optimal antibiotics” were defined as those having established activity against the bacterial pathogen in question, with clinically appropriate selection, dosing, and duration of treatment in accordance with published guidelines and professional standard of care, as confirmed upon review by the authors. Infectious diarrhea was defined as three or more loose stools in a 24-h period accompanied by other gastrointestinal symptoms such as nausea, vomiting, abdominal cramps, or an oral temperature ≥ 38 °C. Patients for whom either the stool culture or FilmArray GI panel were positive but who did not meet the above clinical criterial for infectious diarrhea at the time of presentation were excluded from the analysis.

### Laboratory methods

The FilmArray GI panel includes the following bacterial pathogens: *Campylobacter* (*jejuni, coli*, and *upsaliensis*), *Clostridium difficile* toxin A/B, *Plesiomonas shigelloides, Salmonella* spp., *Yersinia enterocolitica*, *Vibrio* (*parahaemolyticus, vulnificus*, and *cholerae*), Enteroaggregative *Escherichia coli* (EAEC), Enteropathogenic *E. coli* (EPEC), Enterotoxigenic *E. coli* (ETEC), Shiga-like toxin-producing *E. coli* (STEC) stx1/stx2, *E. coli O157,* and *Shigella*/Enteroinvasive *E. coli* (EIEC) [16, 17]. *Shigella* and EIEC could not be differentiated on the basis of the FilmArray GI panel alone without confirmatory cultures. FilmArray GI panel results that were positive for only viral or parasitic pathogens were excluded, as this study focused on the antibiotic management of bacterial diarrhea. All stool samples where bacterial pathogens were detected by the FilmArray GI panel were reflexed to standard stool cultures. Stool cultures were able to identify the following species: *Yersinia*, *Shigella*, *Salmonella*, *Vibrio*, *Campylobacter,* and *E. coli* O157. Stool cultures containing only normal fecal flora, including all *E. coli* serotypes other than O157, were reported as negative. Testing for *C. difficile* was done by the toxin A/B EIA and PCR two-tier approach.

### Data analysis

Univariate and bivariate statistics were calculated for the variables of interest using Statistical Analysis Software (SAS, Cary, NC, software version 9.4). Assumptions of normal distribution were made initially in the study design phase. In the course of the study it became apparent that the two comparison groups were unequally affected by miscoding and chart duplication, resulting in unequal samples size and variances; therefore, Welch’s rather than Student’s t-test was used where appropriate. Where univariate analysis demonstrated non-Normal distribution of the outcome variables, nonparametric analysis was conducted using the Wilcoxon rank-sum (Mann-Whitney U-test) method. Where both comparison groups included up to 50 observations, exact two-sided test statistics were calculated; otherwise the normal approximation with continuity correction of 0.5 was applied. The study procedures were approved by the GW University Office of Human Research Institutional Review Board.

## Results

**Fig. 1:**
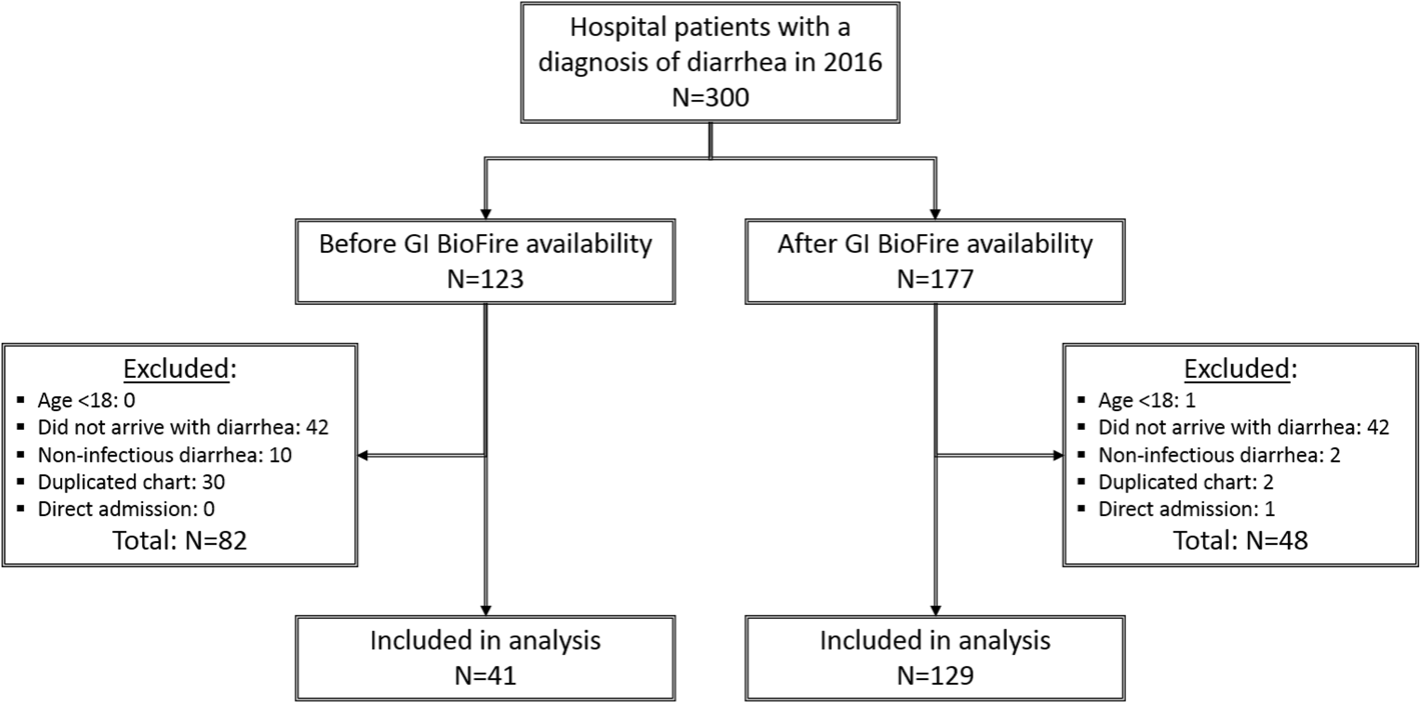
Patient inclusion and exclusion flowchart

Of 300 patient records reviewed for this study, 177 were seen after the introduction of FilmArray GI panel (“FilmArray group”) and 123 were seen before (“pre-FilmArray” or historical control group). Rates of patient exclusion differed between the two groups (Fig. 1). The most common reason for exclusion was lack of diarrhea observed during the hospital stay but not at the time of initial arrival/evaluation (42/177 in the FilmArray group, 42/123 in the pre-FilmArray group), chart duplication (2/177 in the FilmArray group, 30/123 in the pre-FilmArray group), and inclusion of non-infectious diarrhea cases in the initial search results (2/177 in the FilmArray group, 10/123 in the pre-FilmArray group). One hundred twenty-nine of 177 patients (73%) in FilmArray group met inclusion criteria while only 41 of 123 patients (33%) in the pre-FilmArray comparison group met inclusion criteria. This led to an adjustment of the statistical approach as noted in Methods. The resultant groups differed in composition by gender, age, and rates of *C. difficile* infection (CDI) (Table 1).

**Table 1:** Characteristics of patients with suspected infectious diarrhea evaluated before and after introduction of FilmArray GI PCR

Characteristic	Overall	FilmArray group	Pre-FilmArray group	***P***-value*
**Total**				
N	170	129	41	–
**Sex**				
Male – N (%)	79 (46.5%)	65 (50.4%)	14 (34.1%)	**0.0120**
**Age**				
Years – mean ± std. dev.	47.4 ± 18.4	45.6 ± 18.4	56.7 ± 17.9	**0.0011**
**Prior history of CDI**				
Prior *C. difficile* documented in chart – N (%)	22 (12.9%)	13 (10.1%)	9 (22.0%)	**< 0.0001**
**CDI diagnosis (by ICD-9/ICD-10 code)**				
*Clostridium difficile* enteritis/colitis – N (%)	102 (60.0%)	68 (52.7%)	34 (82.9%)	**< 0.0001**
**Time to overall disposition from ED**				
Hours – median [Q1,Q3]	7 [5,10]	7 [5.5,10]	7 [4,10.5]	0.6052
**Time to discharge from ED**				
Hours – median [Q1,Q3]	7 [5,10]	7.5 [5,10]	7 [6,15]	0.5618
**Time to admission to hospital**				
Hours – median [Q1,Q3]	7 [5,10]	7 [6,10]	7 [4,10]	0.4542
**Hospital admission**				
Admitted from ED to hospital – N (%)	117 (68.8%)	81 (62.8%)	36 (87.8%)	**< 0.0001**
**Hospital length of stay (LOS)**				
Days – median [Q1,Q3]	4 [2,8]	3 [2,6]	7.5 [4,10]	**0.0002**
**Day on which empiric antibiotics started**				
Days – median [Q1,Q3]	1 [1,1]	1 [1,1]	1 [1,1]	0.3298
**Day on which optimal antibiotics started**				
Days – median [Q1,Q3]	2 [1,2]	1 [1,2]	2 [1,3]	**0.0117**
**Optimal antibiotics prescribed on first try**				
Discharged with initial antibiotics – N (%)	70 (41.2%)	61 (47.3%)	9 (22.0%)	**< 0.0001**
**Discharged without antibiotics**				
Antibiotics not prescribed or discontinued – N (%)	20 (11.8%)	18 (14.0%)	2 (4.9%)	**< 0.0001**

* Welch’s t-test or Wilcoxon ranked-sum test (Mann-Whitney U-test) for continuous variables, Chi-squared test for categorical variables

**Fig. 2:**
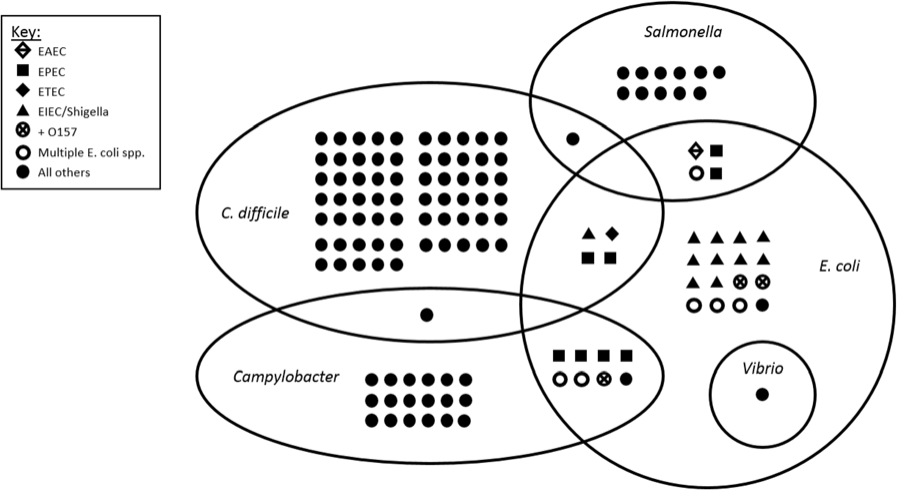
FilmArray GI PCR results as reported on initial stool samples of 129 patients with suspected infectious diarrhea. *E. coli* pathotypes are coded according to the key below, including where two or more pathotypes were detected (○); the three samples in which *E. coli* O157 was detected or co-detected are shown (see text)

**Fig. 3:**
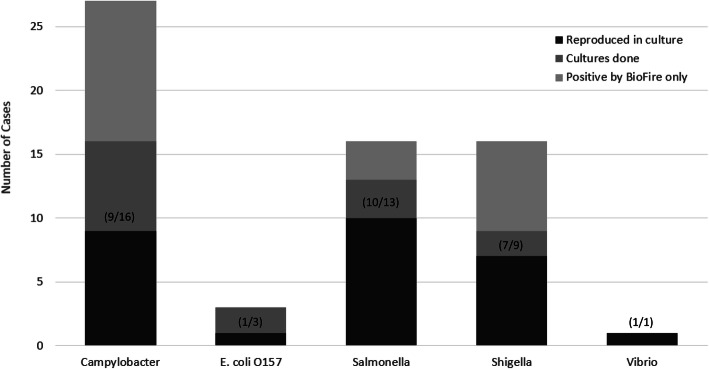
Reproducibility of FilmArray GI PCR findings in culture, by pathogen. Proportion of confirmatory cultures positive for the target organism given in parentheses

Of the 129 patients tested with the FilmArray GI panel, *C. difficile* was the most frequently detected organism and was found in 71 (55.0%) samples. *Campylobacter* was detected in 27 (20.9%) of samples. *Salmonella* was detected in 16 (12.4%) patients, two of whom were bacteremic. BioFire detected *Shigella*/EIEC in 16 (12.4%) patients. EPEC, EAEC, ETEC, and STEC were the most common co-detected organisms, found in 18 (14.0%) of patients, always in combination with other pathogens. There were three cases of *E. coli* O157 detected*,* but only one was confirmed by culture. There was one case of *Vibrio parahemolyticus* detected which was confirmed in culture. Two or more pathogens were detected by FilmArray GI panel in 31 (24.2%) of cases, as illustrated (Fig. 2). Confirmation by culture ranged from 56.3% for *Campylobacter* to 77.8% for *Shigella* (Fig. 3), but was limited by low rates of confirmatory cultures which were only ordered for 40 of the 68 non-*C. difficile* patients.

Median time from ED arrival to overall disposition was 7 h (IQR: 5–10) both for the FilmArray group and the pre-FilmArray group (*p* = 0.6052). This was not significantly different among those patients who were admitted to the hospital or those discharged from the ED. The rate of admission to the hospital was 62.8% in the FilmArray group and 87.8% in the pre-FilmArray group (*p* < 0.0001). The hospital length of stay (LOS) ranged from 1 to 60 days overall, with median LOS of 3 (IQR: 2–6) days in the FilmArray group and 7.5 (IQR: 4–10) days in the control group (*p* = 0.0002).

Median time to initiation of empiric antibiotics did not differ significantly between the two groups, but the time to optimal therapy (defined as the antibiotic prescribed at the time of hospital discharge) was shorter in the FilmArray group (1 vs. 2 days, *p* = 0.0117). The proportion of patients for whom the initial antibiotics choice was not changed for discharge was 47.3% in the FilmArray group vs. 22.0% in the control group (*p* < 0.0001). Patients discharged without antibiotics (either not started on antibiotics, or started and then discontinued prior to discharge) accounted for 14.0% of the FilmArray group and 4.9% of the pre-FilmArray group. Among the 28 patients whose FilmArray GI panel results were confirmed by culture, 28.6% were discharged without antibiotics. All patients diagnosed with CDI received therapy with either metronidazole or oral vancomycin. The most frequent outpatient antibiotic prescribed to patients without CDI in both groups was ciprofloxacin.

## Discussion

The potential of using multiplex PCR testing for the diagnosis and management of infectious diarrheal illness has been extensively documented in a variety of settings [13–15, 17, 20, 21], but so has its potential for yielding results of unclear clinical significance, including false-positives and multiple positives per sample [18, 19, 22, 23]. This tendency is inherent in the technology, and is a result of its reliance on the amplification of genetic loci which may persist in the gut during asymptomatic carriage, or be transferred horizontally between enteric bacteria, particularly *E. coli* pathotypes and other enteric bacteria [16, 17, 22, 24, 25]. In clinical use, this creates a conflict between the desire to expedite diagnostic results in patients with infectious gastroenteritis, and the potential to over-treat non-pathological results.

Co-detection of multiple pathogens, particularly multiple *E. coli* pathotypes, was common in our study. This is similar to findings in prior studies of multiplex PCR panels for gastrointestinal sample analysis, in which co-detections were reported in 16–28% of positive samples [22–26]. The FilmArray GI panel is noted to be more prone to detecting mixed infections than the Luminex xTAG system (Luminex Corporation, Austin, TX), the only other FDA-approved multiplex PCR gastrointestinal panel [22]. Whether such detections represent true coinfections of viable organisms or merely colonization cannot be determined based on these tests alone, and decisions regarding treatment continues to be a clinical issue. Standard bacterial cultures continue to have a key role in confirming PCR diagnoses, but published rates of successful culture confirmation of multiplex PCR findings range widely. In one meta-analysis, culture confirmation was 63.9% for *Campylobacter* (CI: 39.8–84.9%), 48.4% for *Salmonella* (CI: 27.8–69.3%), 73.4% for *Shigella* (CI: 38.1–97.1%), and 75.0% for *E. coli O157* (CI: 50.9–91.3%); overall, the PCR methods detected approximately 1.5 times more pathogens compared to standard stool cultures [18].

Overestimation of *C. difficile* infections is well-documented risk of using PCR-based diagnostics. In one study of the FilmArray GI panel, only 43% of samples where *C. difficile* was detected were subsequently found to produce detectable toxin by enzyme immunoassay (EIA) [27]. In our study we found that physicians did not consistently order a confirmatory EIA in patients who had *C. difficile* detected by the FilmArray GI panel. This has since been addressed with quality improvement interventions in our institution, and a consistent two-step reflex testing algorithm instituted. Physicians have also been educated about refraining from ordering the GI multiplex panel when a high pre-test suspicion for CDI exists. Additional aggressive institutional and regional efforts in antibiotic stewardship and infection prevention aimed at reducing CDI incidence have been ongoing. It is possible that these efforts contributed to the difference in LOS and antibiotics use we observed, though the relatively short period of time captured (2015–2016) makes it less likely that such changes in practice or other secular trends (such as the tendency towards shorter LOS overall) entirely account for our findings.

Several recent multi-center studies have compared the FilmArray GI panel to other PCR assays or conventional culture methods with mixed results. While initial studies showed high sensitivity and specificity of FilmArray GI panel when compared to reference methods [16, 17, 22], other real-world studies have found more variation in the rates of pathogen detection and reproducibility, depending on the practices of specific centers [23–26]. The use of the FilmArray GI panel has implications regarding other aspects of patient care, including use of hospital resources and infection control. Beal et al. found a modest reduction in LOS, days of antibiotics, abdominal imaging studies, and costs per patient associated with the use of the FilmArray GI panel; however, in that study the FilmArray GI panel results were not communicated to physicians in a timely manner, likely reducing their impact on management [28]. In a cost-benefit analysis, the use of the Luminex xTAG gastrointestinal panel was associated with increased diagnostic costs but break-even points and cost savings could be realized contingent on using the test results to reduce isolation days [29]. In practice, physicians frequently fail to follow up on these results with appropriate adjustment of isolation status [30]. Some experts favor restricting the use of multiplex PCR panels to initial evaluations or to patients with severe symptoms in order to improve cost-effectiveness [31, 32].

In our study, the introduction of FilmArray GI panel testing did not appear to affect overall time to disposition (either hospital admission or discharge from the ED), or time to empiric antibiotics. These findings are not altogether surprising, as initial management is more likely to be driven by clinical factors, including patient characteristics and illness severity, than by the findings of a single diagnostic test. Administrative and logistical factors might also affect the time to admission or discharge. Overall LOS was significantly shorter after the introduction of FilmArray GI panel testing, as was the time to initiate appropriate optimal antibiotics. These findings suggest that the use of the FilmArray GI panel as part of the initial evaluation of patients with acute diarrhea of suspected infectious etiology may result in cost savings.

The role of multiplex PCR in antibiotic stewardship remains mixed. On one hand, a positive result in the absence of proper clinical interpretation may lead to unnecessary treatment. The high proportion of patients in our study who received treatment for organisms found on the FilmArray GI panel alone (not confirmed by culture results) may speak to the propensity of physicians to treat reflexively based on reported test results. On the other hand, in the era of stool culture-based diagnosis alone, only 37% of patients in our sample were started on the optimal medication right away, whereas after the introduction of the FilmArray GI panel this increased to 52%, and optimal treatment occurred sooner in their hospital course. By enabling physicians to avoid starting antibiotics where not indicated (e.g. viral enteritis or certain *E. coli* types), and targeting therapy to pathogen earlier in the treatment course, this methodology can support the aims of antibiotic stewardship.

Our study has several limitations. The number of patients meeting inclusion criteria was unequally affected by miscoding (i.e. patient charts initially selected based on diagnostic codes, which were then excluded), resulting in an especially low number in the control group, and compromising the overall statistical power. By its very nature, a retrospective review is dependent upon correct coding, and it is likely that some patients were incorrectly diagnosed and their charts could not be accessed. The high prevalence of CDI-related diagnoses in our pre-FilmArray group may reflect a sampling bias in which cases of suspected infectious diarrhea were not coded as such in the absence of a specific pathogen (in turn reflecting the low diagnostic yield of stool cultures) and thus overlooked when screening patient charts for inclusion in the study. We adjusted for these issues to the best of our ability by using more conservative statistical methods.

## Conclusions

Clinical judgment combined with multiplex PCR and confirmatory stool cultures can provide an approach to infectious gastroenteritis that is both rapid and accurate. The increased laboratory costs associated with this approach should be offset by the reduction in inappropriate antibiotic administration and decrease in LOS. Further research is needed to understand the optimal use and interpretation of multiplex PCR methods in the diagnosis of infectious diarrhea.

## Data Availability

The datasets used and analyzed for this study are available from the corresponding author upon reasonable request. The diagnostic methods used are available from the manufacturer as specified in the text.

## Abbreviations

CDI: *Clostridium difficile* infection
EAEC: Enteroaggregative *E. coli*
ED: Emergency Department
EIEC: Enteroinvasive *E. coli*
EPEC: Enteropathogenic *E. coli*
ETEC: Enterotoxigenic *E. coli*
GI: Gastrointestinal
GW OHR IRB: George Washington University Office of Human Research Institutional Review Board
GWUH: George Washington University Hospital
LOS: Length of stay
PCR: Polymerase chain reaction
STEC: Shiga toxin–producing *E. coli*
